# Utilizing immunogenomic approaches to prioritize targetable neoantigens for personalized cancer immunotherapy

**DOI:** 10.3389/fimmu.2023.1301100

**Published:** 2023-12-12

**Authors:** Ravi K. Shah, Erin Cygan, Tanya Kozlik, Alfredo Colina, Anthony E. Zamora

**Affiliations:** ^1^ Department of Medicine, Medical College of Wisconsin, Milwaukee, WI, United States; ^2^ Department of Microbiology and Immunology, Medical College of Wisconsin, Milwaukee, WI, United States

**Keywords:** cancer immunotherapy, immunogenomics, neoantigens, genomics-based approaches, single-cell technologies, immunogenic neoepitopes, predicting TCR antigen specificity, personalized medicine

## Abstract

Advancements in sequencing technologies and bioinformatics algorithms have expanded our ability to identify tumor-specific somatic mutation-derived antigens (neoantigens). While recent studies have shown neoantigens to be compelling targets for cancer immunotherapy due to their foreign nature and high immunogenicity, the need for increasingly accurate and cost-effective approaches to rapidly identify neoantigens remains a challenging task, but essential for successful cancer immunotherapy. Currently, gene expression analysis and algorithms for variant calling can be used to generate lists of mutational profiles across patients, but more care is needed to curate these lists and prioritize the candidate neoantigens most capable of inducing an immune response. A growing amount of evidence suggests that only a handful of somatic mutations predicted by mutational profiling approaches act as immunogenic neoantigens. Hence, unbiased screening of all candidate neoantigens predicted by Whole Genome Sequencing/Whole Exome Sequencing may be necessary to more comprehensively access the full spectrum of immunogenic neoepitopes. Once putative cancer neoantigens are identified, one of the largest bottlenecks in translating these neoantigens into actionable targets for cell-based therapies is identifying the cognate T cell receptors (TCRs) capable of recognizing these neoantigens. While many TCR-directed screening and validation assays have utilized bulk samples in the past, there has been a recent surge in the number of single-cell assays that provide a more granular understanding of the factors governing TCR-pMHC interactions. The goal of this review is to provide an overview of existing strategies to identify candidate neoantigens using genomics-based approaches and methods for assessing neoantigen immunogenicity. Additionally, applications, prospects, and limitations of some of the current single-cell technologies will be discussed. Finally, we will briefly summarize some of the recent models that have been used to predict TCR antigen specificity and analyze the TCR receptor repertoire.

## 1 Introduction

Cancer significantly impacts human health and quality of life, and is one of the leading causes of death worldwide, with approximately 10 million deaths in 2020, according to WHO (1). Gene mutations caused by genomic instability during carcinogenesis (2), viral infections (3), alternative splicing (4), and gene rearrangements (5) can alter the protein coding regions of the genome resulting in aberrant proteins that are not found in normal cells. On occasion, these protein variants may be processed into small peptides by a tumor cell’s proteasome and bind the major histocompatibility complex (MHC) molecules with sufficient affinity to serve as novel tumor antigens (i.e., tumor neoantigens) that can then be recognized by CD4+ or CD8+ T cells and elicit an antitumor response (6, 7). Collectively, the repertoire of peptides that are displayed on the surface of tumor cells are referred to as the immunopeptidome (8) and each neoepitope (neoantigen bound to a specific MHC molecule) can be recognized by a collection of TCRs resulting in neoantigen-specific TCR repertoires of varying diversity. Neoantigens resulting from missense or fusion mutations aren’t expressed by healthy cells making these neoantigens safe targets for T-cell based immunotherapies due to the ability to generate robust T cell responses and decreases the likelihood of off target toxicity. If neoantigens play a significant role in T cell-mediated tumor resolution, one would posit that tumors with higher mutational burdens would have correspondingly greater frequencies of neoantigen-specific T cells. Along these lines, several clinical trials have explored whether tumor mutational burden and T cell infiltration correlate with efficacy of immune checkpoint blockade (ICB) and/or adoptive cell therapy (ACT) (9–12). These clinical investigations have shed light on the crucial link between tumor neoantigens and the efficacy of immunotherapeutic strategies. Tumors with higher mutational burdens have been associated with greater responsiveness to ICB, such as anti-PD-1 or anti-CTLA-4 antibodies, as the abundance of mutations has been associated with an increased likelihood of there being immunogenic neoantigens that can be recognized by infiltrating T cells, resulting in improved antitumor immune responses. A positive correlation exists between the tumor mutational burden (TMB) and the abundance of neoantigen-specific T cells within the tumor microenvironment, resulting in an elevated rate of response to immunotherapeutic interventions for some cancers (13). Despite these cases, it is noteworthy that low TMB can still give rise to neoantigen-reactive lymphocytes, particularly in hematological malignancies and specific epithelial cancers such as gastrointestinal cancers (14–16). More recently, personalized vaccinations in the form of mRNA, peptides, or peptide-loaded antigen presenting cells (APCs) have been shown to be safe, immunogenic and capable of generating durable clinical response (17–19). Although the tumor mutational burden is highly variable across different types of cancer, immunogenic neoantigens (i.e., neoantigens that induce T-cell activation and proliferation) have been identified in several cancer types, implying that although a patient might have a lower tumor mutational burden (and consequently fewer presented neoantigens) they may still derive benefit from ICB and personalized immunotherapies (20, 21). Also, the majority of tumor neoantigens are private (i.e., unique to an individual rather than being present across the population), suggesting that future decisions regarding the best cancer therapy will need to be made on a case-by-case basis. To date, neoantigens present the most promising target for cell-based immunotherapies; however, the challenges associated with their identification requires adoption of reliable high-throughput neoantigen discovery pipelines to expedite the time it takes to manufacture a personalized cancer immunotherapy. This review will provide a comprehensive overview of currently used methods for identification and validation of candidate immunogenic neoantigens. Initially, we will cover the workflows for identifying candidate neoantigens using genomics-based methods. Next, we will review screening and validation approaches for the candidate neoantigens using antigen-directed and TCR-directed approaches. Furthermore, we will provide overview of reporter methods used to identify neoantigen-TCR pairs and introduce some of the novel single cell platforms that provide a more granular picture of how immune cells respond to tumors. Additionally, to bridge the experimental and computational realms, this review will also showcase the advancements in computational tools for predicting TCR antigen specificity. We will describe some of the innovative bioinformatics approaches and machine learning algorithms that leverage genomics data and experimental insights to predict TCR-pMHC interactions and identify potential target antigens.

## 2 Genomics-based neoantigen identification

Advances in high-throughput sequencing technologies, including greater sequencing speeds and higher accuracy, combined with significant reductions in sequencing cost have enabled researchers to identify an increasing number of somatic mutations and convert the mutated variants to putative neoantigens. The price for genetic sequencing has declined at an astonishing rate, with per genome costs dropping from $50,000 in late 2000 to roughly $600 in 2022 – the price of genome sequencing has recently been predicted to drop as low as $100 per genome with the development of new high-throughput, low-cost sequencing platforms (22). The most common method for identifying putative neoantigens comes from sequencing DNA mutations and/or corresponding RNA. Additional approaches, such as eluting peptides bound to MHC (class I or II) molecules, do not require sequencing data, but these approaches have been covered at length in recent reviews (23–26). Whole genome sequencing (WGS) and whole exome sequencing (WES) combined with RNA-seq are the most widely used approaches for neoantigen discovery, each having their own advantages and limitations. WGS and WES have traditionally been the methods of choice for variant identification. WGS has the advantage of allowing for identification of variants in non-coding regions such as untranslated regions (UTRs) which are missed by WES approaches, but WGS is considerably more expensive than WES due to the extra sequencing coverage required. WES, on the other hand, provides a fine balance between cost and benefits (27) – although this sequencing approach only targets approximately 2-3% of the whole genome, it provides data on the protein-coding regions believed to be the major source of somatic mutations. Combining RNA-seq with WGS or WES provides an additional layer of resolution by incorporating gene expression levels into the process of selecting genes that are more likely to produce translated proteins and filter out candidates that do not meet a predefined threshold. Moreover, other types of neoantigens such as gene fusions, alternative splicing isoforms, and RNA editing events can be revealed from RNA-seq data (28, 29). Although PCR methods have been considered the gold standard for human leukocyte antigen (HLA) typing, RNA-seq data can be used as an alternative given the recent improvements in prediction power now reaching levels comparable to PCR-based approaches (30, 31). Recently, an alternative approach to traditional mRNA sequencing, Ribo-Seq (32), has allowed for rapid identification of neoantigens by providing a snapshot of only the mRNA bound by ribosomes, which depicts all proteins being translated at the time of cell lysis and allows for identification of an expanded set of open reading frames (33, 34). Ribo-seq provides an RNA-sequencing based readout of mRNA translation by isolating ribosome bound RNA fragments, thereby offering a genome-wide footprint of ribosome-RNA interactions. As such, this approach circumvents the experimental difficulties of working with protein molecules and readily identifies translations which might have been missed by other methods. The advantage of Ribo-seq technology in predicting targetable neoantigens is that it identifies only those variants likely to generate proteins and it also provides a more reliable estimation of protein expression. However, it should also be noted that not all the translations reported by Ribo-seq will actually result in expressed proteins and a wide range of functional scenarios may be possible for Ribo-Seq ORF’s (35) including making stable proteins, mediators of gene regulation and having medical implications.

The growing number of peptide:MHC (pMHC) neoepitopes that have been validated using traditional wet lab assays, as outlined below, has resulted in tremendous advancements in the field of neoantigen discovery, and have allowed new computational algorithms to be developed that more accurately predict which peptides bind to specific HLA molecules. While many of these bioinformatic pipelines rely on the same series of steps to prioritize targetable neoepitopes, namely filtering sequence quality, reference mapping, somatic mutation calling, mutated peptide sequence identification, and peptide ranking based on cellular processing and presentation pathways, the accuracy in neoepitope prediction is significantly impacted by the quality and quantity of information contained in the training sets used to develop these algorithms.

### 2.1 Identification of somatic mutations (variants)

Most sequencing efforts have focused on the identification of in-frame insertions and deletions (indels), out of frame insertions and deletions (frameshift mutations), and single nucleotide variants (SNVs), with much less attention given to gene fusions, gene inversions, and gene duplications despite these mutations accounting for a significant number of the mutational landscape and potentially serving as neoantigens with greater immunogenicity. Non-SNV mutations have recently been shown to account for 15% of all neoantigens (36). Somatic variant identification is one of the most critical steps in any pipeline focused on neoantigen discovery as samples from tumor and matched non-tumor DNA sequencing data provides a list of all targetable neoantigens uniquely present in tumor samples. **Table 1** lists some of the widely used mutation callers. It should be noted that NGS sequencing depth directly influences the reproducibility of variant detection; specifically, the higher the number of aligned sequenced reads, the higher the confidence to base call at a specific position, regardless of whether the base call is the same as the reference or mutated nucleotide. Also, higher sequencing depth achieves more sensitive detection of variants at low allele frequency. Numerous studies have directly compared the performance of somatic variant callers (SVCs) (51–53). Somatic variant callers are challenged by the need to balance between accurately identifying true low-allelic somatic mutations and the stringency of the calling procedure to reduce the number of false positive calls. Because of disparities in the number of mutations predicted by different calling algorithms, selection of an SVC is an important component of neoantigen prediction. Running multiple SVC algorithms simultaneously and using the consensus results can result in improved specificity for the variant detection (54). Consensus approaches present a trade-off as they often improve the specificity at the cost of sensitivity, as the increased specificity will decrease the number of false positives to be tested, but decreased sensitivity could result in missing clinically relevant variants. Regardless of which variant caller or approach is used to predict mutations, it is always recommended to validate the putative mutations by manually reviewing the matched tumor-normal samples in Integrated Genomics Viewer (IGV) (55).

**Table 1:** List of Bioinformatics tools used for variant calling and gene fusion detection.

Tools	Approach/Method	URL	Type of call	Reference
*Small Variants*				
EBCall	Allele Frequency Analysis	https://github.com/friend1ws/EBCall	SNV, Indel	(37)
Mutect	Allele Frequency Analysis	https://github.com/broadinstitute/mutect	SNV	(38)
Strelka	Allele Frequency Analysis	https://github.com/Illumina/strelka	SNV, Indel	(39)
Varscan2	Heuristic Threshold	http://varscan.sourceforge.net/	SNV, Indel	(40)
SomaticSniper	Joint Genotype analysis	https://github.com/genome/somatic-sniper	SNV	(41)
Virmid	Joint Genotype analysis	https://sourceforge.net/projects/virmid/	SNV	(42)
VarDict	Heuristic Threshold	https://github.com/AstraZeneca-NGS/VarDict	SNV, Indel, Structural Variants	(43)
SnooPer	Machine Learning	https://sourceforge.net/projects/snooper/	SNV, Indel	(44)
SomaticSeq	Machine Learning	https://github.com/bioinform/somaticseq	SNV	(45)
*Structural Variants*				
DELLY	Paired end, read depth and Split-read analysis	https://github.com/dellytools/delly	Indels, duplications, inversions and translocation	(46)
Pindel	Paired end and split-read analysis	https://github.com/genome/pindel	Indels, Large deletions	(47)
*Gene Fusions*				
STAR-Fusion	STAR Alignment, artifact filtering	https://github.com/STAR-Fusion/STAR-Fusion	Gene fusion	(48)
Arriba	STAR alignment, read and event level filtering	https://github.com/suhrig/arriba	Gene fusion, Structural variant	(49)
defuse	Paired end, split read alignment, filtering	https://github.com/amcpherson/defuse	Gene fusion	(50)

In addition to SNVs, there has been increasing demand for the development of tools that can provide proper identification of other neoantigen sources including large INDELs (46, 47) and gene fusions (48–50). Structural variants, typically large indels (with or without frameshift) and gene fusions can be identified using a single tool and do not benefit from the consensus approach (56).

### 2.2 Variant annotation

Variant annotation is the process of labeling the variants with genomic or genetic characteristics which can be categorized or prioritized for further investigation. Variant annotation is critical for neoantigen prediction as the mutations can impact the corresponding amino acid sequence and may result in silent variants, missense mutations, frameshifts, mutations in non-coding regions and gain or loss of stop codons, each of which can result in neoantigens with varying immunogenicities. Variant Effects Predictor (VEP) (57) and ANNOVAR (58) are two common variant annotation software programs and have been compared directly using the same set of transcripts (59). Although it is difficult to accurately benchmark the success of different programs, in the comparison study VEP aligned more consistently with manually curated variants. However, ANNOVAR and VEP are command line driven and Perl based tools which can be inherently complicated for researchers without programming backgrounds to use. To interactively annotate the data without programming knowledge, a new R shiny based interactive application called ShAn (60) was developed and has demonstrated greater speed and online accessibility compared to VEP and ANNOVAR with comparable predictive capabilities.

### 2.3 Antigen processing algorithms

While neoepitope prediction algorithms have resulted in tremendous advancements in our ability to predict which neoantigens will likely bind to specific HLA molecules, greater accuracy can be achieved when upstream events involved in antigen presentation are incorporated. It is widely known that peptide-pulsing studies that rely solely on MHC-binding algorithms tend to overestimate the number of immunogenic neoantigens that would be naturally processed and presented due to the fact that not all full-length proteins will be cleaved by the proteasome or immunoproteasome and result in peptides with mutated amino acids being loaded onto specific MHCs (61, 62). Before MHC class I presentation, peptides are transported by the transporter associated with antigen processing (TAP) protein to the endoplasmic reticulum (ER) and then trimmed by ER-related aminopeptidases (ERAP) present there (63). There are several tools that predict both proteosomal cleavage and account for TAP’s peptide transportation efficiency. For MHC class I processing and presentation, NetChop20S (64) and ProteaSMM (65) have been shown to reliably predict *in vitro* cleavage patterns owing to the large proteasome digestion training sets used to train these models (66). For MHC class II (MHCII) processing and presentation PepCleaveCD4 (67) and MHCII-NP (68) are two tools that predict antigen excision positions resulting in epitopes that can be recognized by CD4+ T cells. While these tools have generally improved the accuracy of neoepitope prediction pipelines, a major limitation is that genes coding for proteins of various components of the antigen presentation machinery, such as TAP1, TAP2, B2M, are known sites of mutation in cancer. Therefore, the utility of these algorithms in identifying cancer neoepitopes needs to be evaluated on a case-by-case basis.

The affinity of peptide to a given MHC molecule is an important contributor to neoantigen immunogenicity and is a major factor that should be weighed by major epitope prediction algorithms. It is also crucial to know the HLA type of the patient before ranking the peptides as it is widely established that different MHC allotypes differ in specificity with respect to peptide binding. State-of-the-art methods currently available to predict peptide presentation are based on artificial neural networks trained on large datasets and are known to perform best on frequent and well-characterized alleles (e.g. HLA-A*02:01) and their accuracy is decreased for rare or not well-studied HLA alleles. Also, HLA gene expression (69) and somatic mutation patterns (70) in this locus need to be evaluated as HLA downregulation or loss of heterozygosity are known mechanisms to disrupt neoantigen presentation and can result in immune escape. Currently, HLA class I typing algorithms relying on NGS data can accurately predict HLA class I alleles with up to 99% (71) accuracy when using WES/RNA-seq data, however HLA class II algorithms are less reliable and require further development to improve their prediction accuracy. Although many class I typing algorithms exist, Optitype (72), Polysolver (73) and PHLAT (74) are the most frequently used and have the highest reported accuracy. More recent algorithms, such as xHLA (75) and HLA-HD (76), have expedited the HLA typing process and show comparable accuracies to those methods mentioned above.

**Figure 1:**
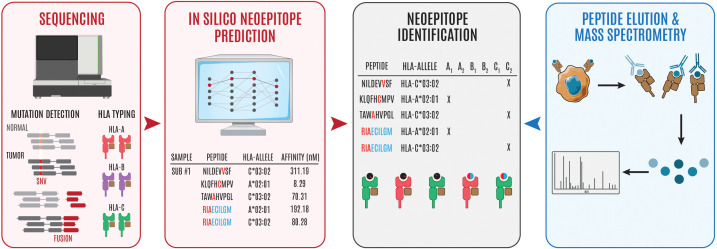
Overview of steps involved in neoepitope identification. Genomics-based neoantien identification begins by performing WGS/WES or RNA sequencing to identify tumor mutations and HLA haplotype, respetively. Following mutational profiling and HLA typing, peptide, MHC prediction algorithms can be used to identify puta- tive neoepitopes (steps in red to black). Alternatively, cancer cells can be isolated and the surface peptide, MHC complexes can be isolated, the peptides can be eluted, and subsequently interrogated using mass spectrometry to identify putative neoepitopes (steps in blue to black). The '#" symbol is used to refer to "number." In this case Sub #1 means "Subject number 1." The "*" symbol is used in standard HLA nomenclature to separate the gene from the allele group.

By combining peptide:MHC binding affinity and HLA typing algorithms or peptide elution and mass spectrometry-based assays, researchers can quickly generate a list of putative neoepitopes (**Figure 1** **).** Several recent reviews (77–82) and articles (83, 84) have provided comprehensive coverage and new considerations when developing computational algorithms for identifying cancer neoantigens. In **Table 2**, we provide an overview of the different genomic-based methods used for neoantigen prediction and describe their advantages and limitations. Incorporation of the antigen processing steps may provide a more accurate depiction of putative MHC-binding peptides; however, most of these algorithms cannot predict whether the bound peptides will be immunogenic (i.e., induce an active response by T cells) (85–87). Ultimately, for a neoepitope to serve as a therapeutic target, it must first be recognized by a cognate TCR and result in a productive TCR signaling cascade. While some MHC class I neoepitope prediction algorithms are beginning to factor in immunogenicity as a parameter for neoantigen prioritization, in most cases robust data sets are still lacking and traditional assays to perturb T cell functionality (e.g. cytokine secretion and/or cytotoxicity assays using peptide-pulsed APCs) are necessary. These approaches are even more essential for MHC class II neoepitopes which are more difficult to predict using in silico algorithms. In **Table 3**, we provide an overview of frequently used neoantigen prediction tools and highlight key features for each.

**Table 2:** Genomic and transcriptomic-based methods for identifying candidate neoantigens.

Methods	Advantages	Limitations
Whole Genome Sequencing (WGS)	Identifies variants in non-coding regions, providing comprehensive genomic information	Costlier due to the requirement for extra sequencing coverage, potentially leading to higher expenses.
Whole Exome Sequencing (WES)	Balances cost and benefits by targeting protein-coding regions, capturing essential genomic information at a more manageable expense	Targets only 2-3% of the genome, potentially missing variations in non-coding regions that could be biologically significant.
RNA-seq and Alternative Approaches	- RNA-seq reveals gene fusions, alternative splicing, and RNA editing events, offering insights into post-transcriptional regulation. - Ribo-seq provides a snapshot of mRNA bound by ribosomes, offering a genome-wide footprint of ribosome-RNA interactions.	- Not all translations reported by Ribo-seq may result in expressed proteins, necessitating careful interpretation. - Different functional scenarios are possible for Ribo-Seq open reading frames (ORFs), requiring detailed analysis.
Somatic Variant Identification	- Critical for neoantigen discovery by providing a list of targetable neoantigens. - Running multiple somatic variant callers improves specificity.	- Balancing between accurate identification and reducing false positives is challenging. - Consensus approaches trade off sensitivity for specificity, impacting the comprehensiveness of variant calling.
Variant Annotation	- Critical for categorizing variants for further investigation, aiding in prioritization.	- Difficult to benchmark the success of different programs, making it challenging to determine the optimal annotation strategy for specific analyses.
Antigen Processing Algorithms	- Incorporates upstream events in antigen presentation for greater accuracy. - Predicts proteasomal cleavage and TAP’s peptide transportation efficiency.	- Limitations in predicting mutations in genes involved in the antigen presentation machinery, potentially leading to incomplete assessments.
Peptide : MHC Binding Affinity	- Affinity to MHC molecules is crucial for neoantigen immunogenicity. - Artificial neural networks offer accurate predictions for frequent alleles.	- Accuracy decreased for rare or not well-studied HLA alleles, emphasizing the need for further advancements in prediction models to encompass a broader range of human leukocyte antigen (HLA) diversity.

**Table 3:** List of commonly used bioinformatics tools for neoepitope prediction.

Tool	Key features	References
NetMHC/NetMHCpan	-Predicts peptide-MHC binding affinity - Widely used tool for MHC binding prediction -NetMHCpan covers a broad range of MHC alleles	(88–90)
MuPeXi	-Extracts mutated peptides and predicts MHC binding affinity -Integrates multiple tools for a comprehensive analysis	(91)
pVAC-Seq	-Identifies and prioritizes neoantigens based on mutation data - Considers MHC binding, transcript expression and clonality	(92)
INTEGRATE-Neo	-Integrates RNA-seq and DNA sequencing data for immunogenic neoantigen prediction	(93)
NeoPredPipe	-Predicts neoantigens considering MHC binding, T-cell receptor recognition and gene expression -Multiparameter prediction approach	(94)
NetCTLpan	Predicts CTL epitopes based on MHC-I binding, proteasomal cleavage and Tap transport efficiency -comprehensive approach for CTL epitope prediction	(95)
MHCflurry	Deep learning-based tool for predicting peptide-MHC binding affinity	(96)

## 3 Screening and validation steps to identify neoantigen-specific T cells

### 3.1 Antigen-directed approaches

Antigen-directed approaches have rapidly gained prominence as a method for identifying antigen-specific T cells. Early approaches to determine T cell reactivity against neoantigens included culturing T cell clones with target cells containing the appropriate HLA molecules and transfected with tumor complementary DNA (cDNA) library pools (97, 98). This approach has resulted in the identification of several well-known antigens including MAGE (99) and MART-1 (100). Serological analysis of recombinant cDNA expression helped identify NYESO-1 (101), another widely known tumor antigen. However, the major limitation of these approaches is that they require large numbers of neoantigen-specific T cells to perform the screens and to validate immunogenic neoantigens, which may be a limiting factor if cells obtained from biopsies or clinical specimens are scarce.

Another common neoantigen-specific T cell screening approach is to use pMHC tetramers (102) or multimers, which are oligomers formed from four (tetramer) or more (multimer) MHC subunits containing the neoantigen of interest. While tetramers are often sufficient to stain T cells with high-affinity TCR:pMHC interactions, multimers are often necessary for detecting TCRs that bind pMHC with lower affinities (KD < 10 μM) and avidities (103, 104). Although laborious to produce, as unique multimers must be generated for every MHC:peptide combination, they have been used to identify thousands of antigen-specific T cells across several studies (104, 105). For cases where greater avidity between the pMHC and TCR are necessary, pentamers (106, 107) or dodecamers (108) can be used in common immunological workflows such as flow cytometry. To improve on the throughput of tetramer-binding assays, T cell responses against large peptide libraries can be screened using UV-sensitive peptides (109) or thrombin cleavage (110) of peptide linkers which allows for the production of thousands of different monomeric molecules from the same pMHC molecule. Nevertheless, to overcome the limitations imposed by cellular input from clinical biopsies, additional multiplexed and combinatorial tetramer staining approaches are needed. With combinatorial staining, a specific T cell population is identified using tetramers containing the same peptide but conjugated to multiple fluorochromes rather than just a single fluorochrome. Using this approach with just two different fluorochromes per peptide drastically increases the number of specificities that can be examined simultaneously from a single sample using flow cytometry (e.g., if each peptide:MHC specificity is identified using a dual fluorochrome approach and a total of 8 fluorochromes are used across antigen specificities, this will result in a two-dimensional matrix capable of identifying up to 28 unique specificities) (111). While this approach significantly increases the number of antigen specificities that can be screened from a single sample, other conditions may be limiting, such as the configuration of flow analyzers and the number of fluorochromes incorporated in phenotypic panels to robustly characterize antigen-specific T cells with sufficient resolution (i.e., with low enough spillover). To overcome this limitation, mass spectrometry can be used as an alternative, in which tetramers are labelled with isotopically purified metal conjugates providing little spillover between labels, less variation in signal intensity between parameters, and many more surface markers to include phenotypic characterization of neoantigen-specific T cells (112, 113). Although mass-spec based approaches such as CyTOF address the issue of multiplexing, cellular material cannot be recovered from these workflows as cells are incinerated and impossible to retrieve for downstream applications. More recently, the use of DNA barcoded pMHC multimers has extended this toolkit (114). While the increased capability of labelling multimers with DNA barcodes offers flexibility to screen entire cancer mutanomes in one single reaction, it only provides information about the frequency of corresponding T cells but lacks the inclusion of functional readouts as the cells are lysed for sequencing. An upgraded version of DNA barcoded multimers called tetramer-associated TCR sequencing (tetTCRSeq) (115) can further link the antigen specificity to the TCR sequences in single cells at high throughput. This technology allows for the simultaneous generation of peptide libraries and DNA barcodes using *in vitro* transcription and translation (IVTT), thereby reducing labor and cost but still allowing the recall of antigen specificity and TCR sequences.

Although pMHC multimers have revolutionized the field of neoantigen discovery, screening approaches using multimers require prior knowledge of the antigens being targeted (i.e., these approaches aren’t completely unbiased and not truly *de novo* screening approaches). Additionally, pMHC multimers aren’t commercially available for every HLA molecule, which has been a major impedance in the field. Moreover, the low accuracy of in silico prediction of HLA class II-restricted epitopes and technical issues with the production of pMHCII multimers possess challenges for screening and identifying neoantigens presented by MHCII molecules. Furthermore, TCRs that bind pMHC tetramers with low affinity can be difficult to detect by flow cytometry and may not accurately reflect the affinity needed for T cell activation, which can underestimate the frequency of neoantigen specific T cells. Other potential bottlenecks attributed to the use of pMHC multimers include limited throughput of peptide synthesis, maximum library size, instability of pMHC multimers, and PCR amplification bias.

With recent advances in microfluidics, additional tools to study TCR:pMHC interactions with higher throughput have started to emerge. Microfluidic approaches can be divided broadly into three categories (1): trap-based devices (2), valve-based devices, and (3) droplet microfluidics. In this review we will mainly focus on droplet microfluidics for single cell analysis, but we would highly encourage readers to read a recent review that provides additional coverage of additional approaches (116). Droplet microfluidics separates individual cells into low volume oil droplets, making precise characterization of complex immune responses possible at single cell resolution. Since this technique has minimal sample loss and requires very low cell input, having a limited number of cells from tumor biopsies or clinical specimens doesn’t exclude this approach from being used for screening applications. Building on droplet microfluidics, the nuclear factor of T cells (NFAT)-eGFP reporter system was used for functional screening and real-time monitoring of T cell activation kinetics upon recognition of tumor cells, enabling quick identification of the responding T cells and verification of corresponding TCR sequences that could then be used as therapies for cancer (117). In a similar study, nanoparticle barcoded nucleic acid cell sorting (NACS) (118) was used to count and isolate neoantigen specific T cells. MATE-Seq (119), another technique that builds on the approaches used in NACS (118), allowed for high-throughput isolation and single cell TCR sequencing of neoantigen specific T cells using magnetic nanoparticle-barcoded pMHC tetramers linked to photocleavable TCR-specific primers. These antigen-directed approaches can simultaneously increase the number of putative neoantigens that can be screened and identify TCRs capable of binding, but they often underestimate immunogenicity due to the lack of a functional readout.

### 3.2 TCR-directed approaches

To overcome challenges associated with peptide-pulsing using individual peptides per condition, which requires high cellular input, and the limitations of peptide-MHC binding predictions for specific HLAs, a new screening assay was designed which allowed T cell responses to any non-synonymous mutation across HLAs to be evaluated (120). For this approach, each mutation is expressed by a single minigene designed to encode the mutated amino acid flanked by 12 amino acids of the wildtype sequence on both sides. Tandem minigenes (TMG) were generated by stringing together 6 to 24 minigenes in a single open reading frame, *in-vitro* transcribing to generate mRNA, and then transfecting the mRNA into autologous APCs. As an alternative to the TMG screening approach, peptides (25 amino acid residues in length) can be synthesized and pooled together to generate peptide pools (PP). Pools for all putative neoantigens can be screened using either the TMG approach, which utilizes the natural antigen processing and presentation machinery (APPM) of a cell, or by peptide pulsing, which bypasses the APPM and instead directly binds surface MHC molecules. Once T cell reactivity is detected, either through cytokine secretion or upregulation of a specific activation marker (e.g., 4-1BB), against a specific pool of TMGs or PP, further deconvolution of each immunogenic neoantigen found within the pool of reactive peptides can be screened individually. This unbiased approach has been used to identify multiple mutated antigens and neoantigen specific TILs that have been shown to induce antitumor responses in patients (85, 120–122). The biggest advantage of using TMGs and peptide pools is that it mimics processing and presentation of neoepitopes on both class I and II HLA molecules without bias, overcoming the need for in silico prediction and enabling the identification of neoantigen-reactive CD4 T cells since the prediction algorithms for HLA class II molecules aren’t fully optimized. Although in theory one could screen every mutation identified by NGS using TMGs and/or using peptide pools, in practice, this isn’t feasible due to the cost associated with peptide synthesis and the time required to carry out *in-vitro* transcription, especially in cases for tumors with high mutational burdens. As with the tetramer-binding assays, the deconvolution steps needed to identify the specific immunogenic neoantigens requires large numbers of target and effector cells which can be limiting for clinical specimens. To compensate for the lack of autologous APCs, HLA matched cells or monoallelic antigen presenting cell lines can be generated. Alternatively, cell-free antigen presenting beads, generated by coupling the peptide-HLA complex of interest with costimulatory antibodies on the surface of microbeads can be used to determine T cell reactivity (123, 124). Even with the limitations surrounding the number of cells needed to adequately perform these screens, these approaches allow for unbiased identification of candidate neoantigens without prior knowledge of whether the corresponding peptides are capable of binding patient specific HLAs.

### 3.3 Reporter systems to identify neoantigen-TCR pairs

Novel reporter systems have recently been described using mammalian cell surface display, which results in a detectable signal as soon as a cognate TCR binds to a cancer neoepitope presented by an APC. Signaling and antigen-presenting bifunctional receptor (SABR) (125) is a cell-based platform for T-cell antigen discovery that relies on screening of large numbers of antigens through expression of chimeric receptors consisting of an extracellular pMHC complex fused to an intracellular CD28 costimulatory and CD3ζ signaling domain in NFAT-GFP Jurkat cells. Upon recognition by a TCR, the CD28-CD3ζ signaling triggers the expression of GFP in APCs for the identification of the presented peptide by downstream sequencing. This technology has been demonstrated to successfully identify the cognate antigen for TCRs from a large library of epitopes and for the discovery of personalized neoantigens.

Another system, T-Scan (126), is a high-throughput platform for systematic identification of antigens recognized by T cells that incorporates an engineered reporter for granzyme B activity by tagging APCs expressing epitope coding minigenes. Upon recognition of antigen by cognate TCR, granzyme B secreted from the T cells leads to the reconstitution of a fluorescent reporter in the APCs. Utility of T-scan was validated by showing that it can identify known peptide epitopes of both viral and human-genome libraries (126). Another similar system leveraging the specificity of the granzyme-perforin pathway (127), used a reporter-fusion protein consisting of cyan fluorescent protein (CFP) and Yellow Fluorescent protein (YFP) separated by a peptide linker harboring a granzyme B recognition site. Upon recognition of target cells by T cells, cleavage of the fusion protein by granzyme B causes a loss in the fluorescence resonance energy transfer (FRET) signal generated from YFP and results in a concomitant gain of CFP signal that is easily identifiable and allows for the isolation of recognized target cells by fluorescence-activated cell sorting (FACS).

Another similar system based on the NFAT reporter was developed to identify tumor specific CD4 T cells by fusing murine MHCII with the signaling domains of TCR resulting in generation of pMHC-TCR (MCR) hybrid molecules (128). MCR libraries were generated by cloning tumor cDNA into MCR sequences and transducing reporter cells which can then be used as APCs in multiple rounds of coculture with T cells. Upon interaction of specific TCR with the MCR, NFAT activation results in the expression of a reporter gene, which ultimately allows the activated reporter cells to be identified, single cell sorted by FACS, and the recognized peptides can be identified by sequencing the corresponding DNA.

Lastly, trogocytosis, which is the transfer of membrane protein from one cell to another was used to identify the sequence of peptides (129). Upon successful interaction of TCR expressing Jurkat cells and pMHC presenting K562 cells, transfer of TCR from the Jurkat cells to K562 cells can be detected by identification of K562 cells with TCR using FACS. Following cell sorting of K562 cells containing transferred TCRs, PCR amplification and deep sequencing is used to identify the corresponding epitope of interest.

**Table 4** succinctly summarizes some of the most common methods, assays, and techniques that can be used to screen and validate neoantigen specific T cells.

**Table 4:** Common methods, assays, and techniques that can be used to screen and validate neoantigen specific T cells.

Approach Type	Methods/Techniques
**Antigen-Directed Approaches**	- Utilization of pMHC tetramers or multimers for precise detection of T cells with high-affinity TCR:pMHC interactions. - Screening large peptide libraries using UV-sensitive peptides and thrombin cleavage. - Combinatorial staining employing multiple fluorochromes to enhance specificity. - Adoption of mass spectrometry for high-throughput analysis with minimal spillover. - Integration of DNA barcoded pMHC multimers for flexibility and high throughput. - Leveraging droplet microfluidics for single-cell analysis with minimal sample loss
**TCR-Directed Approaches**	- Implementation of Tandem minigenes (TMG) and peptide pools for unbiased screening of T cell reactivity. - Functional assays measuring T cell reactivity against TMGs or peptide pools and application of deconvolution techniques to identify specific immunogenic neoantigens. - Use of autologous APCs, HLA-matched cells, or cell-free antigen-presenting beads for enhanced flexibility in screening.
**Reporter Systems**	- Utilization of SABR and T-Scan systems, incorporating chimeric receptors and measuring granzyme B activity for high-throughput T-cell antigen discovery. - Integration of NFAT reporter systems for the identification of CD4 T cells. - Application of Trogocytosis for TCR transfer, facilitating the identification of specific peptides involved in immune responses.

## 4 Single-cell platforms

**Figure 2:**
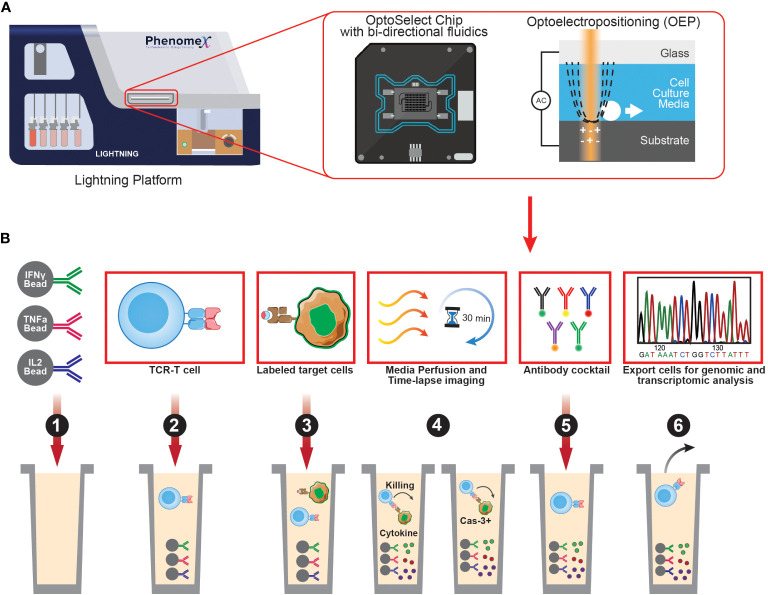
Overview of PhenomeX's optofluidic platform and multi-parameter workflow. **(A)** Overview of PhenomeX's Lightning platform, OptoSelect Chip, and Optoelectroposition. **(B)** As a first step (step 1) for the multi-parameter workflow, OptoSelect chips are loaded with cytokine capture beads of interest. Then (step 2), T cells are single cell sorted into nanopen chambers. Next (step 3), labeled target cells are single cell sorted into nanopen chambers containing T cells. Media is then perfused and time-lapse images (step 4) are recorded throughout the duration of the co-culture experiment. Following the co-culture, antibodies can be added (step 5) to target surface markers or captured cytokines. Lastely (step 6), cells of interest can be exported for downstream assays (e.g., transcriptional profiling or continued off-chip expansion).

Single-cell sequencing is a powerful tool that enables high-throughput analysis of genetic or transcript material for single cells. One of the most widely used platforms for this purpose is the Chromium system by 10X Genomics, which can sequence both the transcriptome and TCRs of single cells by incorporating cell barcodes. While scDNA-seq can reveal mutations and structural changes in cell genomes, it is not as frequently utilized due to limited DNA copies available in single cells, making scRNA-seq a more popular option for measuring gene expression across multiple transcripts. It is important to note that many single-cell sequencing technologies currently only allow assessment of up to 10,000 cells, which is several orders of magnitude lower than conventional bulk sequencing methods. Nevertheless, scRNA-seq can be leveraged to identify multiple layers of information such as cell phenotype using oligo-tagged antibodies in combination with transcriptome. In comparison to bulk RNA-Seq approaches, scRNA-seq provides more detailed and comprehensive characterization of individual cells. Recent advances in single-cell platforms have enabled additional multiplexing capabilities to interrogate neoantigen-specific T cells. The PhenomeX Lightning and Beacon platforms combine optics and fluidics to link phenotypic, functional, and transcriptomic profiles to single neoantigen-reactive T cells (**Figure 2** **) (** 130). These technologies can characterize and isolate single cells from a larger population of cells that display desired phenotypes and/or functional characteristics. There are three main features that make the PhenomeX’s optofluidic platforms uniquely suited for antigen discovery. First, at the core of the technology is the OptoSelect Chip which enables isolation of single cells using Opto-electropositioning (OEP) and nanopen chambers. OEP functions to guide cells in and out of selected nanopens using a non-destructive localized light-induced electric field that can move cells using a weak repulsive force against charged membranes or particles. This feature can be used to sort or “pen” single antigen-presenting cells encoding a library of neoepitopes and a single T cell in each nanopen, avoiding the need to set up bulk coculture experiments as required for earlier assays. Second the platform contains fluidics capable of bi-directional flow through the chip which can function to help transfer single cells into nanopens, diffuse media throughout the chip during culturing assays, and also recover specific cells of interest from the chip. Third is the optics system which acts as a microscope and fluorescent analyzer and allows for brightfield and fluorescent images to be acquired, cell counts to be made and phenotypic and functional assays to be performed using fluorescent readouts. By modifying these assays, multiple parameters can be assessed in single nanopens to assess T cell activation upon recognition of cognate pMHC complexes (e.g., similar to T-scan, capture beads for Granzyme B can be added in each nanopen). Upon successful interaction of T cell and pMHC expressing APCs, Granzyme B secreted by neoantigen-specific T cells can be captured by capture beads, and subsequently stained by a fluorescently labeled anti-granzyme B antibody binding a different epitope. Alternatively, additional functional readouts such as IFN-gamma or IL-2 secretion can also be assessed. Future studies using PhenomeX’s platforms could also provide screening workflows to test libraries of T-cell clones and pMHC simultaneously as the user has greater control over the loading of specific cells into distinct nanopens on the chip, expanding the number of effectors and target cell pools that can be screened at once. Depending on the required throughput, either the Lightning (for more validation-based workflows requiring 1,500 nanopens or less) or the Beacon (for either validation- or discovery-based workflows requiring greater than 1,500 nanopens) platform could be used. However, further advancements in the development of fluidics-based methods would be required to dissect the T-cell specificity at larger scales and to scale up to those routinely performed in bulk coculture assays. Finally, desired cells recovered from these platforms can be sequenced using single-cell sequencing, providing a comprehensive understanding of the genetic and transcriptomic features of individual cells of interest and their roles in complex biological processes.

In the field of cancer immunotherapy, single-cell technologies have begun to take center stage and have helped lead to a greater appreciation of the dynamic interactions between cancer and immune cells. A notable advancement involves the concurrent evaluation of T cell receptor (TCR) sequences and gene expression profiles from individual T cells, highlighting the intricate connections between T cell functionality and gene expression within the tumor microenvironment. Recent studies have shed light on how the TCR repertoire significantly influences the functional attributes of individual T cells (131–135), which can help guide newer approaches for developing cell-based therapies for cancer. Previous challenges in properly identifying rare and elusive cell types within the TME, such as cancer stem cells or immunosuppressive cell subsets, have also been addressed with single-cell technologies by empowering researchers to isolate and analyze these rare cell types with unprecedented precision and resolution. Techniques like single-cell genomics and proteomics provide key insights into how these cells drive tumor progression, metastasis, and therapy resistance. By performing scRNA-seq on tumor biopsies, the corresponding transcriptional profiles of individual cells can be used to identify global and local signatures (136–140), and determine whether specific subsets of cells, such as effector T cells, regulatory T cells, dendritic cells, and macrophages, have infiltrated specific regions of a tumor. This high-resolution analysis provides crucial insights into the functional states, activation statuses, and potential immunosuppressive features of distinct immune cell populations, which if used on longitudinally collected samples, can also help determine whether a patient is responding to a specific therapeutic intervention. The seamless integration of single-cell technologies propels cancer immunotherapy into a realm of precision and depth previously unattainable, promising a future where therapeutic strategies are finely tuned to each patient’s immune landscape.

## 5 Advances in computational tools for prediction of TCR antigen specificity

As the number of high throughput workflows for sequencing and TCR functional characterization have increased, several databases such as McPAS-TCR (141), VDJdb (142), and the TBAdb subset of PIRD (143) have emerged as repositories for TCR and epitope information. Building upon the unprecedented amount of experimental data generated by single-cell platforms, recent advances in digital biology and machine learning have expanded our ability to predict T-cell receptor (TCR) antigen specificity and analyzing TCR repertoires. While traditional approaches like X-ray crystallography, Nuclear Magnetic Resonance (NMR), Surface Plasmon Resonance (SPR) and Mass Spectrometry (MS) are still used for confirming conformational and structural interactions (144–148), they are limited by the time-consuming process of characterizing pMHC-TCR interactions one by one. However, cutting-edge approaches that leverage computational tools and machine learning algorithms are bridging the gap between experimental data and predictive modeling, offering new possibilities for understanding immune responses and developing personalized immunotherapies.

These innovative tools analyze the sequence and structural features of TCRs and their corresponding antigen epitopes, establishing patterns and predicting potential interactions. They can be broadly classified into two categories: supervised predictive models (SPMs) and unsupervised clustering models (UCMs) (149) based on their use of supervised and unsupervised learning, respectively. Arsenal of tools have emerged recently that uses the training data set generated by the experimental approaches to general a model. Some of the widely used tools have been summarized in **Table 5**.

**Table 5:** List of bioinformatics tools for TCR repertoire analysis and TCR-pMHC specificity prediction.

Tool	Description	Key Features	Availability	URL	References
GLIPH	Cluster TCR sequences based on shared motifs	Motif based clustering, identification of hotspots	Open-source software, Web based service	https://github.com/immunoengineer/gliph	(150, 151)
TCRdist	Measures similarity between TCR sequences based on CDR motifs	Repertoire wide TCR distance calculation	Python Package	https://github.com/phbradley/tcr-dist https://tcrdist3.readthedocs.io/en/latest/	(152, 153)
IGoR	Estimation of TCR repertoire diversity	Probability of CDR3 generation, V(D)J recombination	C++ package	https://github.com/qmarcou/IGoR	(154)
TCRex	Deep learning based framework for TCR specificity prediction	Integration of diverse TCR features, deep learning	Web based tool	https://tcrex.biodatamining.be/	(155)
DeepTCR	Deep learning based tool for antigen-specific TCR prediction	Integration of TCR sequence and binding data	Python Package	https://github.com/sidhomj/DeepTCR	(156)

One widely used tool is GLIPH (150), which applies clustering techniques to identify functionally related TCRs by identifying shared binding motifs in their CDR3 sequences. By uncovering these motifs, GLIPH and its successor GLIPH2 (151) enable the inference of potential epitopes and TCR-pMHC interactions. Another commonly employed tool, TCRdist (152, 153), calculates distances between TCR CDR3 sequences, providing a measure of similarity and aiding in the inference of antigen specificity. IGoR (154) is an additional tool frequently utilized in the field, estimating the probability of generating a specific CDR3 sequence through V(D)J recombination. By accounting for the recombination process, IgoR offers a more accurate estimation of TCR repertoire diversity and antigen specificity, enhancing the understanding of the T cell repertoire and the likelihood of eliciting a specific TCR response. TCRex (155), another prominent tool, is a computational framework trained on large-scale TCR-pMHC binding datasets that employs deep learning algorithms to predict TCR-pMHC interaction specificity, facilitating the identification of potential target antigens. Furthermore, DeepTCR (156), a deep learning-based tool, analyzes TCR repertoires to predict antigen specificity of TCRs and identify potential epitope targets. By integrating experimental data, computational techniques, and machine learning algorithms, these tools have revolutionized TCR sequencing, enabling researchers to rapidly predict potential targets.

These innovative tools and approaches have not only provided a solid foundation for understanding basic biological principles surrounding TCR repertoire diversity and the influence of pMHC on this diversity but also hold great potential for transforming personalized therapies and clinical care. Their utilities extend to fields such as transplantation (GVHD), autoimmunity (cross-reactivity), and immunotherapy (cell-based therapies and cancer vaccines), offering valuable insights and paving the way for personalized immunotherapies.

## 6 Concluding remarks

Neoantigens have emerged as promising targets in cancer immunotherapy. In silico identification of candidate neoantigens is a critical step in personalized immunotherapy that relies on the development and application of bioinformatics and computational approaches. Neoantigen prediction algorithms have continued to evolve and improve in recent years, which has been aided by the ever-growing training data sets. However, more robust measures are needed to improve the identification of immunogenic neoantigens, as not all predicted neoantigens elicit an immune response. Screening and validation of neoantigen specific T cells using antigen- or TCR-directed approaches provides greater efficiency and accuracy in identifying targetable neoantigens, but they are expensive and time consuming. The recent advances in single cell technologies have offered new ways to integrate multiple information layers at the level of individual cell. While these technologies offer exciting prospects, they also present challenges in data analysis and interpretation. Additionally, the prediction of TCR-pMHC interactions plays a vital role in translating neoantigen into actionable targets for cell-based therapies. Cutting edge approaches, that leverage the computational tools and machine learning algorithms, have been developed recently offering new possibilities for understanding immune responses and developing personalized immunotherapies. Overall, these innovative tools, in combination with experimental data, pave the way for more effective and tailored immunotherapeutic approaches, offering valuable insights for personalized immunotherapies.

## Author contributions

RS: Conceptualization, Writing – original draft, Writing – review & editing. EC: Writing – review & editing. TK: Writing – review & editing. AC: Writing – review & editing. AZ: Writing – review & editing, Conceptualization, Funding acquisition, Visualization, Writing – original draft.
